# Correction: Perceptions of healthcare finance and system quality among Nigerian healthcare workers

**DOI:** 10.1371/journal.pgph.0005068

**Published:** 2025-08-13

**Authors:** Blessing Osagumwendia Josiah, Emmanuel Chukwunwike Enebeli, Brontie Albertha Duncan, Lordsfavour Uzoma Anukam, Oluwadamilare Akingbade, France Ncube, Chinelo Cleopatra Josiah, Eric Kelechi Alimele, Ndidi Louis Otoboyor, Oghosa Gabriel Josiah, Jemima Ufuoma Mukoro, Blessing Chiamaka Nganwuchu, Fawole Israel Opeyemi, Timothy Wale Olaosebikan, Marios Kantaris

In the Abstract section, there is an error in the third and 11th sentences of the paragraph. The correct third sentence is: A cross-sectional survey was conducted with 584 healthcare professionals from nine States across Nigeria, representing a variety of healthcare occupations. The correct 11th sentence is: By implementing these reforms, Nigeria can enhance the quality and accessibility of healthcare services and improve the health and well-being of its citizens and residents.
