# Supplementary material for: Perceptions of healthcare finance and system quality among Nigerian healthcare workers
Source: PLOS Glob Public Health. 2024 Nov 7;4(11):e0003881. doi: 10.1371/journal.pgph.0003881 (PMC11542805; doi:10.1371/journal.pgph.0003881)
Supplement: S1 Table — provides a breakdown of the professional designations of the 584 Nigerian health workers who responded to this survey. Majority of the respondents are involved in clinical roles, with nurses and midwives being the most represented profession. However, non-clinical roles such as administration, cleaning, and data management are also significantly represented. (DOCX) [file pgph.0003881.s002.docx]

S1 Table: Professional Designations of Respondents

|  | | Professional Designation | | Total |
| --- | --- | --- | --- | --- |
|  |  | Clinical | Non-clinical |  |
| Professional Designation | Nurse and/or Midwife | 183 | - | 183 |
|  | Physician | 74 | - | 74 |
|  | Health Care Assistant | 56 | - | 56 |
|  | Pharmacist | 41 | - | 41 |
|  | Laboratory Scientist | 33 | - | 33 |
|  | Physiotherapist | 22 | - | 22 |
|  | Radiographer | 13 | - | 13 |
|  | Imaging (Radiographer, Sonographer etc.) | 12 | - | 12 |
|  | Emergency Medical Service or Ambulance Services | 7 | - | 7 |
|  | Speech therapist | 4 | - | 4 |
|  | Administration or Management | - | 26 | 26 |
|  | Cleaners (Housekeeping) | - | 26 | 26 |
|  | Data management, front desk, records | - | 25 | 25 |
|  | Driver or Security | - | 19 | 19 |
|  | Porter, Kitchen staff, or Maintenance (e.g., Oxygen Team Team) | - | 19 | 19 |
|  | Morgue Attendant (mortuary worker) | - | 10 | 10 |
|  | Social Worker | - | 10 | 10 |
|  | Occupational therapist | - | 4 | 4 |
| Total | | 445 | 139 | 584 |
