# Supplementary material for: Perceptions of healthcare finance and system quality among Nigerian healthcare workers
Source: PLOS Glob Public Health. 2024 Nov 7;4(11):e0003881. doi: 10.1371/journal.pgph.0003881 (PMC11542805; doi:10.1371/journal.pgph.0003881)
Supplement: Attachment — Submitted filename: RESPONSES TO REVIEWER.docx [file pgph.0003881.s003.docx]

**RESPONSES TO REVIEWER #1**

Reviewer #1: Thank you for the opportunity to review this valuable manuscript. The study's objective of evaluating the quality of the health system in Nigeria is of great significance. It provides crucial insights that can greatly benefit policymakers in Nigeria and other countries with similar healthcare infrastructures. Here are some recommendations to enhance the transparency of the study and make it even more informative about improving the quality of the health system.

*Response:* We appreciate your kind encouragement and have therefore taken the time to review the entire manuscript in line, and in some instances, beyond with your recommendations. Your valuable comments and suggestions have been addressed as described below.

**Methods:**
Overall, I missed some key information about the data collection tool, the type of health providers included, the level of healthcare providers (primary, secondary, tertiary), the study area, the population, and the data analysis. See specific points below:

*Response*: Our Data were mostly collected from Secondary health facilities across the states. We did not specifically collect data on the specificities of the facilities however, we ensured that all facilities were government-owned. Further research may be required to show any differences in these areas.

1. Did you consider heterogeneity in health worker density across states and urban/rural scenarios to calculate the sample size? For example, Kano has the biggest total population, but Lagos may have a higher density of health workers despite its smaller population. So, would this change the sample size estimate?

*Response*: The numbers obtained initially for healthcare workers in some states were conflicting. For instance, in Oyo states, we initially obtained figures for nurses from the Nurses Union level, Ministry Level, and Hospital Level for similar research, but these figures were not consistent, hence we adopted the National estimate and utilized proportion and respondents’ rates to further normalized the density.

The state-level health workers' density was not taken into consideration during the initial calculations, however, during data collection these differences normalized as we visited facilities within different cities and communities. You may notice in Table 1 that selected participants exceeded the expected samples at different rates from each state, which we believe was partly due to the influence of the existing density of healthcare workers.

2. Can you provide more information about the study population and inclusion criteria, including clinical and non-clinical categories: doctors, nurses, and technicians? The same is true for study settings (urban/rural) and types of health facilities (primary, secondary, tertiary level; public, private; outpatient, inpatient care). Please clarify the inclusion criteria. Also, how did you select the health units and workers in each state?

*Response* Thank you for drawing our attention to this. We have adjusted the population and sampling process report to show the criterion for inclusion.

“Therefore, the target population for the study was 143,835 healthcare workers in addition to some non-clinical healthcare workers who are working in Nigerian government health facilities currently or within the last year (32).”

Furthermore, we added, “The survey was aimed at those with about ten years of experience; hence the respondents' cut-off age was 30 years and above to ensure that they have an average of 5-10 years post-qualification.”

3. It is unclear how the sample of 600 health workers was reached. I understand you tried to capture the difference between clinical and non-clinical staff, but how were these additional figures calculated?

*Response*: Thank you for pointing this out. We have now corrected the sample size determination process report as shown below.

“The sample size was calculated with the formula Z^2*p*q / m^2, {where Z = 1.96 at 95% Confidence Interval, p = 0.5 (incidence rate), q = 0.5 (non–incidence rate) and m = 0.05 (Margin of error)} using a total healthcare workers population of 143,835 across the nine States. The resultant sample of 384, predominantly comprised of clinical healthcare workers, was proportionately selected across the nine states (Table 1). For inclusiveness, a broader range of perspectives, enhanced statistical power, and representativeness in the selected States, non-clinical healthcare workers were added to the sample.

In Nigeria, the proportion of clinical and non-clinical team members is approximately 65% to 35%, respectively, based on a pilot study conducted in Edo and Imo States. This is unlike the composition of the estimated 19.97 density reported by the WHO which significantly excludes the non-clinical staff from the measurement even though they closely interact with the healthcare system and typically make up about half of the healthcare staff (33). An additional sample of 216 (36%) non-clinical workers was, therefore, selected during the survey to increase the initial sample size to a target of 600 participants (Table 1).

4. Include more information about the data collection tool; for example, was it based on previous studies? Also, include information on how many health workers were included to test the questionnaire's reliability.

*Response*: Thank you for the kind observation, we have now addressed this point as shown below.

Data was collected via a structured questionnaire in print and online on Google Forms, measuring sociodemographic data, perceptions of healthcare quality, challenges, and areas of improvement as developed based on the Donabedian healthcare quality assessment model (35), the SERVIQUAL framework (36), Mosadeghrad's conceptual framework for quality of care (37) as applied by other similar studies (38), as well as the Patient Satisfaction Questionnaire (PSQ-18) which is used for the measurement of patient satisfaction with the medical care received (39). It was composed of both yes or no questions and a Likert scale that measured the perceptions of healthcare quality. A reliability test was conducted with two samples of 25 healthcare workers from Imo and Edo States respectively with a reliability coefficient (Cronbach alpha) of 0.70.

5. Can you please justify using the Pearson correlation and which correlation you are looking at? The same is true for regression analysis. Which regression did you use, which variables (exposure and explanatory), and why was this test applied? It seems that regression is more useful in finding associations, but the article states that it was applied to ‘exploring variations in perceptions across demographics’. I could not find these ‘variations’ in the results section.

*Response*: Thank you for the corrections, a review of the analysis has been done. We have adopted the use of Chi-squared and Regression analysis for the respective variables and amended the discussions accordingly. We acknowledge also that the work has undergone a major correction thereby appreciating your valuable comments.

**Results:**
1. Did you find any differences in satisfaction across states or even between clinical and non-clinical professionals? It would be an interesting finding to explore.

*Response*: The new analysis reflects the differences across sociodemographic factors, including state. We also tested for professions as recommended. The findings are indeed very interesting. Thanks for your recommendation as shown on Table 6 to Table 11.

1. Elaborate more on the description of Table 3: include frequencies and percentages and the average percentage for each grade (E, G, F, P, VP). Also, Include frequencies in this Table.

*Response*: Thank you so much for the suggestions, we have adopted this approach and included frequencies in each table as required.

2. Include labels in Figures 3 and 4.

*Response*: Thank you for this observation. Labels have now been included in Figure 3 and Figure 4 as pointed out. Also, we revisited the data and revamped the analysis significantly.

2. This sentence should be in the methods section: ‘In the study’s Likert scale evaluation of the Nigerian healthcare system, ratings were assigned as follows: 5 for Excellent, 4 for Good, 3 for Fair, 2 for Poor, and 1 for Very Poor. The cumulative scores of participants were then categorized into ranges: 6-10 for Very Poor, 11-15 for Poor, 16- 20 for Fair, 21-25 for Good, and 26-30 for Excellent.’

*Response*: Thanks for the suggestion. We have now adopted this style and rewritten our data analysis process as shown below.

“The survey was conducted between 8/6/2023 and 20/8/2023 and the data was collated with Google sheet and transferred to Microsoft Excel and SPSS version 29 to be analyzed. The Likert scale evaluation of the Nigerian healthcare system, ratings were assigned as follows: 5 for Excellent, 4 for Good, 3 for Fair, 2 for Poor, and 1 for Very Poor. The cumulative scores of participants were then categorized into ranges: 6-10 for Very Poor, 11-15 for Poor, 16- 20 for Fair, 21-25 for Good, and 26-30 for Excellent. Simple frequency distributions and percentages were applied to the sociodemographic data and various variables. Multiple linear regression analysis and Chi-square test were used to evaluate relationships between the workers’ healthcare quality and system ratings and several factors like professional designations, clinical designations, type of employment, the States, and other sociodemographic attributes. The variations within each State were not examined due to the unified approach to data collection at the State level.”

3. There is no description of Figure 3.

*Response*: Thank you for pointing this out. We have now included the descriptions for each Figures and Tables.

4. Please clarify what Figure 2 represents. For example, what do you mean by satisfaction?

*Response*: We apologize for the clumsiness of our Figure 2 caption. We have addressed this and relocated it to a more appropriate point in the manuscript.

5. The description of the Pearson correlation seems to be misinterpreted. The correlation of 0.5 shows that there is a ‘moderate’ positive correlation between rating and satisfaction, not that satisfaction tends to increase alongside higher ratings.

*Response*: We have now readdressed our analysis and utilized different statistical tests for the categorial and ordinal data for clarity. We have also shown the full tables of each inferential analysis on Tables 6 to 11 for ease of reference.

6. Overall, I could not understand the regression analysis, mainly because of the lack of information in the methods section. Can you clarify the variables you applied in Table 6? For example, what is the reference category for each variable?

*Response*: Thank you once again and apologies for the incomplete expressions in our result presentations. We have now eliminated the previous Table 6 and reintroduced a clearer approach to the test of associations using Chi-square, Simple crosstabs, and further regression analysis as required.

**Discussion:**
This section needs a little more work. At the moment, each paragraph often summarises the findings from this study and then references other studies elsewhere. Still, the link between the two isn’t always explicit, and the implications for the Nigerian health system aren’t always stated. See more details below.

*Response*: We are sorry for the disjunct in our discussions. We had done the discussion over using an adjusted analysis from our source data. Most of the corrections below this point may longer apply.

1. The statement’ The potential skew towards nursing priorities due to their overrepresentation is noted’, indicates that it is important to include this variable in Table 2. Also, this statement pertains to the limitations section.

*Response*: We have now corrected this statement in line with your recommendation. Skewness was removed from the expression to avoid ambiguity.

Perceived Quality of Healthcare:

3. Why would an increase in government healthcare subsidies translate to improvement in healthcare worker's perspective? What is this statement based on? How about the other improvements? Why have they not been reflected in improving the quality of the health system?

*Response*: We have adjusted this portion as well to reflect the new analysis. Thanks for the corrections. Kindly review our new discussions which is now better structured, coherent, and elaborate.

4. Insufficient training was reported as a long-term issue for poor ratings. However, increasing the number of training centres was one of the improvements reported (~51%), which contradicts this statement.

*Response*: Thank you for this remark. The increase in the number of training centers is still not sufficient to meet the rising demands of the national healthcare systems. There is still a gap that is being identified by the healthcare workers and even the government as highlighted in the recent National Health Care Workforce Policy. The new discussion clarifies this.

5. Patient perception is not the focus of this study, so how can you state that there is a discrepancy between patients and health workers' perceptions?

*Response*: Thanks for the observation. Part of the data from healthcare budgets, healthcare workers’ perception, and the patients’ perceptions were initially analyzed together thereby creating that error of interference. We have made the necessary changes to it.

Most prominent challenges

6. Can you include more details of the previous studies when you mention them: setting, focus (specific health conditions or healthcare facilities)?

*Response*: We have now included more details and context for each study cited. Thank you for the correction.

7. Table 3, not Figure 2, highlights the most critical challenges in Nigerian healthcare as identified by healthcare workers.

*Response*: Yes, Figure 3, not Figure 2. This has now been corrected and moved as necessary.

8. Brain drain was not reported in the results section, but the discussion says that 88.7% of respondents reported it as an issue. Please include it in the results.

*Response*: Thank you for drawing our attention to this. It must have been taken from the parent data from the patients’ aspects. It is now corrected.

Public Health Implications and Policy Recommendations

9. This section brings several recommendations, all very important and with an important impact on improving the health system's quality. However, more reflection on the feasibility of implementing all these policies/interventions in a lower-middle-income country would make this section more beneficial from the policy perspective.

*Response*: Thank you for this valuable comment. We have amended our recommendations based on the corrected analysis and discussions.

**Conclusions**
The study's focus on the lack of appropriate funding overshadows the other challenges reported with similar frequency. I recommend reflecting on the overall challenges and how these findings can guide the changes the health workers want to see across the health system in Nigeria.

*Response*: Thank you for this valuable comment. We have amended our conclusion based on the corrected analysis and discussions.
